# The value of T1 mapping and intravoxel incoherent motion parameters in predicting PD-L1 expression and dynamically monitoring immunotherapy in advanced lung cancer

**DOI:** 10.3389/fonc.2026.1687192

**Published:** 2026-02-13

**Authors:** Qijia Han, Shulin Li, Zhiming Xiang

**Affiliations:** 1 Department of Radiology, The Affiliated Panyu Central Hospital, Guangzhou Medical University, Guangzhou, China; 2 Postgraduate Cultivation Base of Guangzhou University of Chinese Medicine, Panyu Central Hospital, Guangzhou, China

**Keywords:** magnetic resonance imaging, T1 mapping, intravoxel incoherent motion, lung cancer, immunotherapy, quantitative evaluation

## Abstract

**Purpose:**

To investigate the value of T1 mapping and intravoxel incoherent motion diffusion-weighted imaging (IVIM-DWI) quantitative parameters in predicting PD-L1 expression and dynamically monitoring immunotherapy efficacy in advanced lung cancer.

**Materials and methods:**

23 patients pathologically diagnosed with advanced lung cancer were collected. Patients underwent T1 mapping and IVIM examinations pre- and post-immunotherapy. The relationship between PD-L1 expression levels and quantitative parameters was analyzed. The changes of quantitative parameters during treatment were compared between the effective and ineffective groups. The predictive value of quantitative parameters for immunotherapy efficacy was analyzed using area under the curve (AUC).

**Results:**

In terms of predicting PD-L1 expression, there were no significant differences in the T1, apparent diffusion coefficient (ADC), perfusion fraction (f), true diffusion coefficient (D), and pseudo-diffusion coefficient (D*) values between the positive and negative groups (all *p*>0.05). After immunotherapy, the effective group showed a lower T1 value (*p* = 0.008) and a higher ADC value (*p* = 0.013), with slight increases in f, D, and D* values. The ineffective group had slight increases in T1 and D values, and slight decreases in ADC, f, and D* values. Compared to the ineffective group, the effective group had a lower post-treatment T1 value (*p* = 0.019) and a higher ΔADC value (*p* = 0.031). The AUCs of the post-treatment T1 and ΔADC values in predicting immunotherapy efficacy were 0.871 and 0.886, respectively.

**Conclusion:**

T1 mapping and IVIM quantitative parameters may reflect potential trends in predicting PD-L1 expression, have certain value in monitoring the dynamic changes during immunotherapy in advanced lung cancer, and are expected to provide meaningful imaging biomarkers for individualized immunotherapy decision-making.

## 1 Introduction

According to the latest statistics provided by the International Agency for Research on Cancer (IARC), lung cancer is the malignant tumor with the highest morbidity and mortality rates in China. Approximately 75% of lung cancer patients have already been in the intermediate or advanced stage at the time of diagnosis and have no chance of surgery (1). In light of this challenging scenario, immunotherapy has emerged as another major breakthrough in the treatment of advanced lung cancer, following the development of radiotherapy, chemotherapy, and molecular targeted therapies. Notably, the research of PD-1/PD-L1 immune checkpoint inhibitors (ICIs) has attracted much attention (2). PD-1/PD-L1 inhibitors exert therapeutic effects by blocking immune checkpoint molecules, thereby suppressing the tumor immune response. This intervention not only improves the quality of life of lung cancer patients but also significantly increases their survival rates. However, the invasiveness of histopathological examination, which is regarded as the gold standard for assessing PD-L1 expression in tumors, remains a critical concern (3). Consequently, there is an urgent need to develop a non-invasive evaluation method to serve as an alternative to conventional biopsies.

Given the individual differences among patients, there are certain differences in the response of different patients to immunotherapy. The Response Evaluation Criteria In Solid Tumors (RECIST) version 1.1 primarily assesses the therapeutic efficacy based on alterations in tumor size and volume; however, it appears to be insufficiently sensitive in immunotherapy. Unlike traditional treatments that directly target tumor cells, immunotherapy stimulates the body’s immune system to attack them. Changes in biological activity may precede observable changes in tumor volume. For this reason, it is imperative to explore a novel non-invasive quantitative approach that accurately captures the microenvironmental dynamic alterations occurring within and surrounding the tumor during immunotherapy. Such an approach would provide clinicians with valuable and real-time feedback, so as to adjust the treatment plans effectively, which is essential for optimizing patients clinical outcomes (4).

Magnetic resonance imaging (MRI), as an advanced non-invasive imaging technique, holds a pivotal position in the realm of tumor diagnosis and treatment, due to its distinctive imaging principles and multi-parameter capabilities. Among its various applications, intravoxel incoherent motion diffusion-weighted imaging (IVIM-DWI) stands out as a bi-exponential model-based technique for assessing the diffusion of water molecules within tissues as well as microcirculatory perfusion (5). Several studies have demonstrated the considerable potential of IVIM in differentiating between benign and malignant lung lesions, distinguishing histological subtypes of lung cancer, assessing lymph node involvement, and predicting therapeutic efficacy (6–8). In addition, T1 mapping, as a quantitative MRI technique, provides an objective assessment of tissue cell characteristics and their extracellular matrix through measuring the T1 relaxation time in tissues (9). Although T1 mapping has shown application promise in distinguishing histological subtypes and differentiation degrees of lung cancer (10–12), to the best of our knowledge, there have been no studies exploring the T1 mapping technique in predicting the efficacy of immunotherapy in lung cancer patients.

Therefore, the aim of this study is to evaluate the value of T1 mapping and IVIM multi-quantitative parameters in predicting PD-L1 expression and exploring the dynamic alterations of these parameters during immunotherapy in advanced lung cancer, so as to enhance the understanding of the underlying mechanisms of immunotherapy, with a view to providing an effective reference for the formulation of personalized treatment strategies and the accurate assessment of the therapeutic efficacy for lung cancer.

## 2 Materials and methods

### 2.1 Patients

Patients pathologically diagnosed with advanced lung cancer at our hospital between August 2021 and June 2022 were prospectively enrolled in this study. Each patient underwent a MRI examination that included T1 mapping and IVIM sequences within one week prior to treatment, and subsequent follow-up MRI scans were performed within 7 days after receiving two cycles of immunotherapy. Post-treatment MRI examinations of all patients were performed strictly in accordance with the above time point to ensure the time consistency of post-treatment evaluation and reduce the impact of confounding factors. The inclusion criteria were as follows: (1) a pathologically confirmed diagnosis of lung cancer; (2) clinical stage IIIB-IV; (3) undergoing PD-L1 immunohistochemistry analysis; and (4) receiving first-line immunotherapy and evaluating the efficacy of the treatment. The exclusion criteria included: (1) concurrent diagnosis of other malignant tumors besides lung cancer; (2) inability to fulfill the diagnostic requirements due to incomplete MRI sequences resulting from insufficient patient cooperation; (3) presence of MRI contraindications; and (4) receiving only one cycle of immunotherapy. The study received approved by the Medical Ethics Committee of our hospital.

### 2.2 MRI acquisition

All the examinations were performed with a 3T MR scanner (MAGNETOM Prisma, Siemens Healthcare, Erlangen, Germany) equipped with a dedicated 18-channel body phased-array coil. Prior to the examination, all patients underwent breath-holding training to ensure optimal image quality. T1 mapping was acquired by breath-holding with the following parameters: repetition time/echo time [TR/TE], 5.24/2.30 ms; matrix size, 224×168; field of view [FOV], 400×300 mm; flip angles [FA], 3°and 15°; slice thickness, 5 mm; and acquisition time [TA], 14 s. IVIM was acquired under free breathing with the following parameters: TR/TE, 4300/50 ms; matrix size, 134×108; FOV, 380×300 mm; slice thickness, 5 mm; TA, 360 s; and 8 b-values (0, 50, 100, 150, 200, 400, 600, and 800 s/mm^2^).

### 2.3 Efficacy evaluation

The efficacy of immunotherapy was evaluated in accordance with the RECIST 1.1 recommended by the international guidelines (13). Specifically, (1) Complete response (CR) refers to the complete disappearance of the target lesion and the short axis reduction of the pathological lymph node by less than 10 mm; (2) Partial response (PR) is defined as the reduction of at least 30% in the total of longest diameters of the target lesions; (3) Stable disease (SD) is classified as a status that falls between PR and Progressive disease (PD); (4) PD is defined as a 20% increase in the sum of the diameters of all measured target lesions during the experimental study. In this study, patients presenting with CR and PR were grouped into the effective group, whereas those presenting with SD and PD were divided into the ineffective group.

### 2.4 Image processing

The region of interest (ROI) was manually delineated by a radiologist with 5 years of experience in pulmonary tumor imaging under the double-blind principle. The largest cross-section of the tumor and the two adjacent layers above and below were selected. During delineation, areas containing major vessels, necrotic tissue, and artifacts were avoided as much as possible. Quantitative parameters, including the apparent diffusion coefficient (ADC), perfusion fraction (f), true diffusion coefficient (D), pseudo-diffusion coefficient (D*) and T1 values, were measured independently for each layer. The average of the three measurements was calculated and recorded as the final value for the patient. All delineations were independently reviewed and verified by a senior radiologist with over 20 years of experience in pulmonary tumor imaging. Any discrepancies were resolved by consensus between the two radiologists.

### 2.5 Statistical analysis

Statistical analysis was performed utilizing SPSS 27.0 statistical software. The Shapiro-Wilk test was used to assess the normality of the measurement data. Data that conformed to a normal distribution were expressed as means ± standard deviations. Independent samples t-test was used when homogeneity of variance was satisfied; otherwise, Welch’s t-test was applied. Paired t-test was employed for inter-group comparisons pre- and post-treatment. Those that did not conform to a normal distribution were expressed as median and interquartile range, using the Mann-Whitney U test for inter-group comparisons or Wilcoxon signed-rank test for pre- and post-treatment comparisons. A *p*-value less than 0.05 was considered to be statistically significant. Additionally, receiver operating characteristic (ROC) curve analyses were employed to evaluate the diagnostic performance of significant parameters, with the area under the curve (AUC) calculated to quantify their discriminative ability.

## 3 Results

### 3.1 General information

A total of 23 patients with advanced lung cancer were enrolled in this study, comprising 16 males and 7 females, range 41~84 years, mean age 62 ± 8 years. There were 12 patients with positive PD-L1 expression (≥ 1%) and 11 patients with negative PD-L1 expression (< 1%). Of the 19 patients who underwent MRI examination after completing two cycles of immunotherapy, there were no cases of CR. 14 patients who experienced PR were categorized into the effective group; conversely, 5 patients (2 experienced PD and 3 experienced SD) were categorized into the ineffective group.

### 3.2 Correlation of quantitative parameters with PD-L1 expression

The T1, ADC, f, and D* values of the positive PD-L1 expression group were found to be higher than those of the negative PD-L1 expression group, while the D value was lower in the positive group compared to the negative group. No statistically significant differences were found in all MRI quantitative parameters between the PD-L1 positive and negative groups (*p* = 0.211, *p* = 0.976, *p* = 0.260, *p* = 0.079, and *p* = 0.667, respectively) (**Table 1**).

**Table 1:** Correlation of quantitative parameters with PD-L1 expression.

Parameters	PD-L1 expression		t/Z	p
	Positive (n=12)	Negative (n=11)		
T1 (ms)	1916.60 (1709.70, 2308.98)	1839.50 (1669.70, 1906.35)	-1.249	0.211 ^b^
ADC (×10^–6^ mm^2^/s)	1506.90 (1276.22, 1666.00)	1450.20 (1255.45, 1802.70)	-0.030	0.976 ^b^
f (×10^-3^%)	142.60 (119.02, 166.85)	109.00 (99.90, 158.85)	-1.125	0.260 ^b^
D (×10^–6^ mm^2^/s)	1210.19 ± 232.16	1250.39 ± 206.90	0.437	0.667 ^a^
D* (×10^–5^ mm^2^/s)	844.15 (740.75, 898.55)	685.60 (631.20, 787.50)	-1.755	0.079 ^b^

^a^represents independent samples t-test; ^b^represents Mann-Whitney U test.

### 3.3 Comparison of quantitative parameters pre- and post-immunotherapy in the effective group

**Figure 1:**
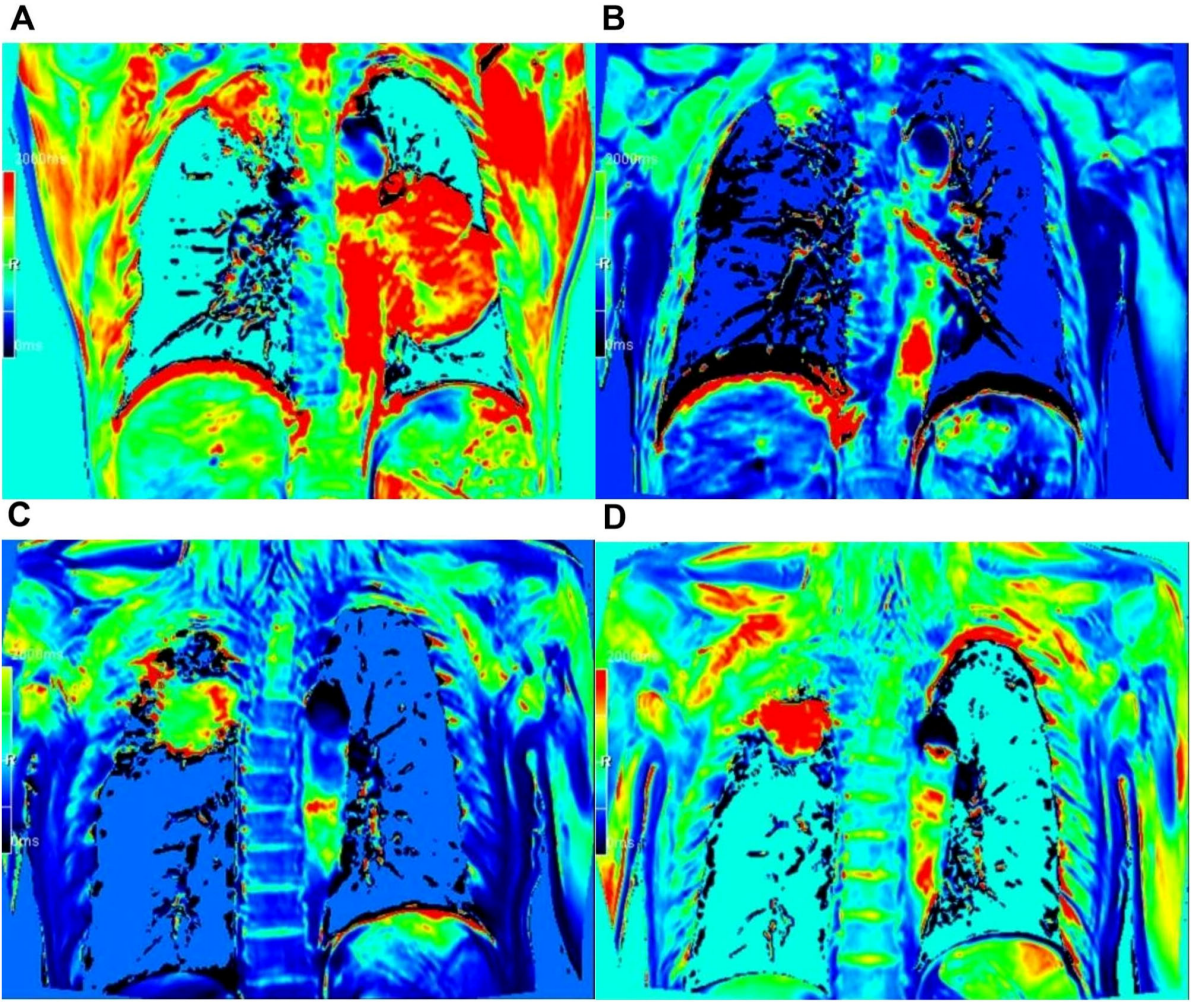
T1 mapping pseudo-color plots pre- and post-immunotherapy in the effective and ineffective groups. **(A, B)** Effective group, male, 68 Y, PR; the post-treatment T1 value was significantly lower than the pre-treatment value, decreasing from 2500.2 ms to 1894.2 ms; **(C, D)** Ineffective group, male, 65 Y, SD; the post-treatment T1 value was higher than the pre-treatment value, rising from 1769.9 ms to 1919.1 ms.

In the effective group, a significant reduction in the post-treatment T1 value was observed compared to the pre-treatment value (1459.54 ± 535.96 ms vs. 1965.02 ± 210.34 ms, *p* = 0.008). In contrast, the post-treatment ADC value exhibited a significant increase relative to the pre-treatment value ((1913.39 ± 468.25 vs. 1425.41 ± 457.03) × 10^–6^ mm^2^/s, *p* = 0.013). Although the post-treatment values for f, D, and D* were higher than their respective pre-treatment values, none of the differences were statistically significant (*p* = 0.074, *p* = 0.729, and *p* = 0.224, respectively) (**Table 2**; **Figure 1**).

**Table 2:** Comparison of quantitative parameters pre- and post-immunotherapy in the effective group.

Parameters	Pre-treatment	Post-treatment	t/Z	p
T1 (ms)	1965.02 ± 210.34	1459.54 ± 535.96	3.109	0.008 ^c^
ADC (×10^–6^ mm^2^/s)	1425.41 ± 457.03	1913.39 ± 468.25	-2.870	0.013 ^c^
f (×10^-3^%)	119.00 (86.60, 183.05)	229.15 (131.60, 371.40)	1.789	0.074 ^d^
D (×10^–6^ mm^2^/s)	1109.09 ± 208.06	1150.37 ± 360.51	-0.355	0.729 ^c^
D* (×10^–5^ mm^2^/s)	825.42 ± 239.07	992.18 ± 382.20	-1.277	0.224 ^c^

^c^represents paired t-test; ^d^represents Wilcoxon signed-rank test.

### 3.4 Comparison of quantitative parameters pre- and post-immunotherapy in the ineffective group

In the ineffective group, the post-treatment T1 and D values were slightly higher than the pre-treatment values. Conversely, the post-treatment ADC, f, and D* values were slightly lower than pre-treatment values. However, these differences did not reach statistical significance (*p* = 0.360, *p* = 0.321, *p* = 0.345, *p* = 0.686, and *p* = 1.000, respectively) (**Table 3**, **Figure 1**).

**Table 3:** Comparison of quantitative parameters pre- and post-immunotherapy in the ineffective group.

Parameters	Pre-treatment	Post-treatment	t/Z	p
T1 (ms)	1808.52 ± 657.40	2114.24 ± 264.63	-1.034	0.360 ^c^
ADC (×10^–6^ mm^2^/s)	1279.80 (1265.50, 1328.30)	1212.90 (1046.40, 2235.65)	0.944	0.345 ^d^
f (×10^-3^%)	139.90 (98.50, 140.50)	117.70 (91.75, 468.25)	0.405	0.686 ^d^
D (×10^–6^ mm^2^/s)	1129.66 ± 119.12	1296.00 ± 313.10	-1.133	0.321 ^c^
D* (×10^–5^ mm^2^/s)	938.56 ± 182.10	938.54 ± 389.48	0.000	1.000 ^c^

^c^represents paired t-test; ^d^represents Wilcoxon signed-rank test.

### 3.5 Comparison of quantitative parameters pre- and post-immunotherapy between the effective and ineffective groups

The pre-treatment T1 and ADC values of the effective group were higher than those of the ineffective group. In contrast, the pre-treatment f, D, and D* values were lower than those of the ineffective group. Nonetheless, these differences were not statistically significant, with *p*-values of 0.426, 0.687, 0.687, 0.839, and 0.352, respectively.

The post-treatment T1 value of the effective group was significantly lower than that of the ineffective group, with a notable difference (1459.54 ± 535.96 ms vs. 2114.24 ± 264.63 ms, *p* = 0.019). The post-treatment f and D values of the effective group were lower, while the ADC and D* values were higher than those of the ineffective group. Nonetheless, none of these differences reached statistical significance, with *p*-values of 0.972, 0.435, 0.226, and 0.792, respectively.

**Figure 2:**
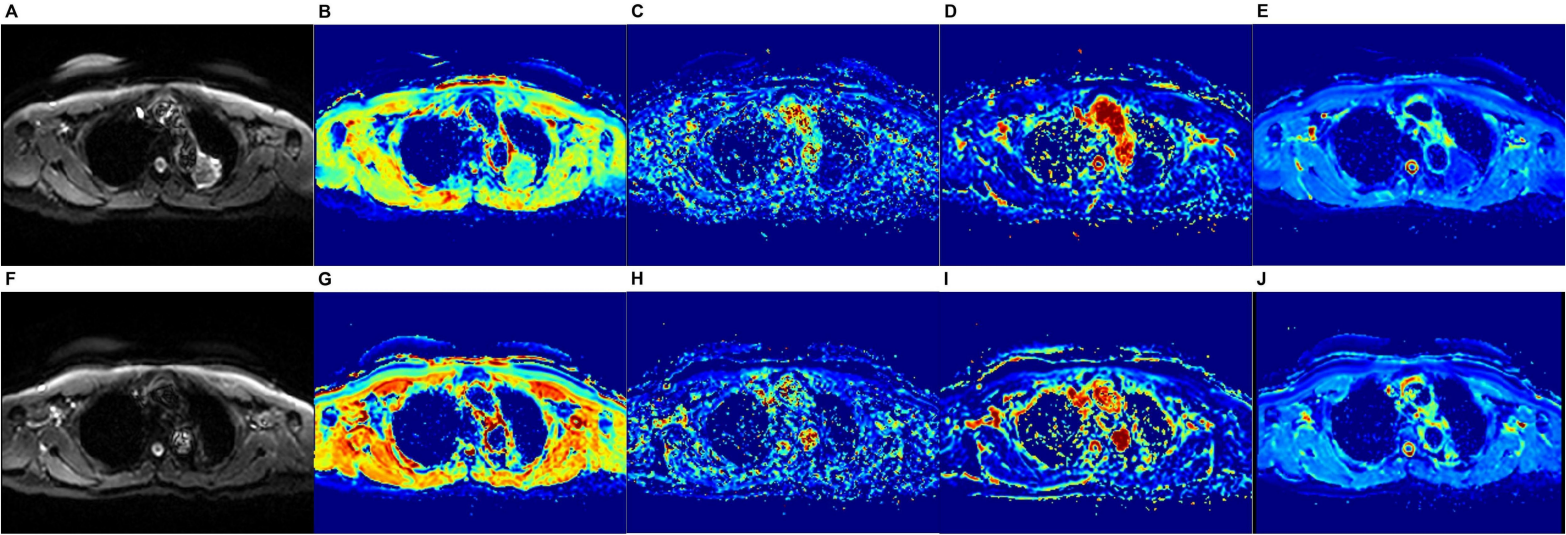
Immunotherapy effective group, female, 68 Y, PR. **(A-E, F-J)** the DWI maps and pseudo-color maps of the D, D*, f, and ADC pre- and post-treatment, respectively. **(A, F)** DWI signal of the tumor was reduced after treatment; **(E, J)** the post-treatment ADC value was significantly higher than the pre-treatment value, rising from 1158.2×10^–6^ mm^2^/s to 2284.6×10^–6^ mm^2^/s; **(B-D, G-I)** the D, D*, and f values did not change significantly pre- and post-treatment.

Furthermore, a comparative analysis of the change in each quantitative parameter pre- and post-immunotherapy revealed that the ΔADC value of the effective group was significantly greater than that of the ineffective group ((516.25 ± 611.19 vs. -190.60 ± 439.83) × 10^–6^ mm^2^/s, *p* = 0.031). The ΔT1 and ΔD values were lower in the effective group compared to the ineffective group, while the Δf and ΔD* values were higher than those of the ineffective group; however, these differences did not reach statistical significance, with *p*-values of 0.056, 0.569, 0.444, and 0.499, respectively (**Table 4**, **Figure 2**).

**Table 4:** Comparison of quantitative parameters pre- and post-immunotherapy between the effective and ineffective groups.

Parameters	Effective group (n=14)	Ineffective group (n=5)	t/Z	p
pre-T1 (ms)	1965.02 ± 210.34	1808.52 ± 657.40	-0.816	0.426 ^a^
pre-ADC (×10^–6^ mm^2^/s)	1293.10 (1108.55, 1600.56)	1279.80 (1265.50, 1328.30)	-0.403	0.687 ^b^
pre-f (×10^-3^%)	119.00 (86.60, 183.05)	139.90 (98.50, 140.50)	-0.403	0.687 ^b^
pre-D (×10^–6^ mm^2^/s)	1109.09 ± 208.06	1129.66 ± 119.12	0.207	0.839 ^a^
pre-D* (×10^–5^ mm^2^/s)	825.42 ± 239.07	938.56 ± 182.10	0.957	0.352 ^a^
post-T1 (ms)	1459.54 ± 535.96	2114.24 ± 264.63	2.586	0.019 ^a^
post-ADC (×10^–6^ mm^2^/s)	1913.39 ± 468.25	1555.40 ± 746.42	-1.257	0.226 ^a^
post-f (×10^-3^%)	244.67 ± 133.25	247.54 ± 206.11	0.036	0.972 ^a^
post-D (×10^–6^ mm^2^/s)	1150.37 ± 360.51	1296.00 ± 313.10	0.799	0.435 ^a^
post-D* (×10^–5^ mm^2^/s)	992.18 ± 382.20	938.54 ± 389.48	-0.268	0.792 ^a^
ΔT1 (ms)	-284.60 (-879.75, -161.23)	92.00 (6.30, 149.20)	-1.913	0.056 ^b^
ΔADC (×10^–6^ mm^2^/s)	516.25 ± 611.19	-190.60 ± 439.83	-2.358	0.031 ^a^
Δf (×10^-3^%)	60.05 (-9.68, 198.68)	-10.90 (-22.20, 9.00)	-0.766	0.444 ^b^
ΔD (×10^–6^ mm^2^/s)	41.29 ± 435.63	166.34 ± 328.30	0.581	0.569 ^a^
ΔD* (×10^–5^ mm^2^/s)	166.74 ± 488.73	-0.02 ± 371.77	-0.690	0.499 ^a^

^a^represents independent samples t-test; ^b^represents Mann-Whitney U test.

### 3.6 Immunotherapy efficacy prediction

**Figure 3:**
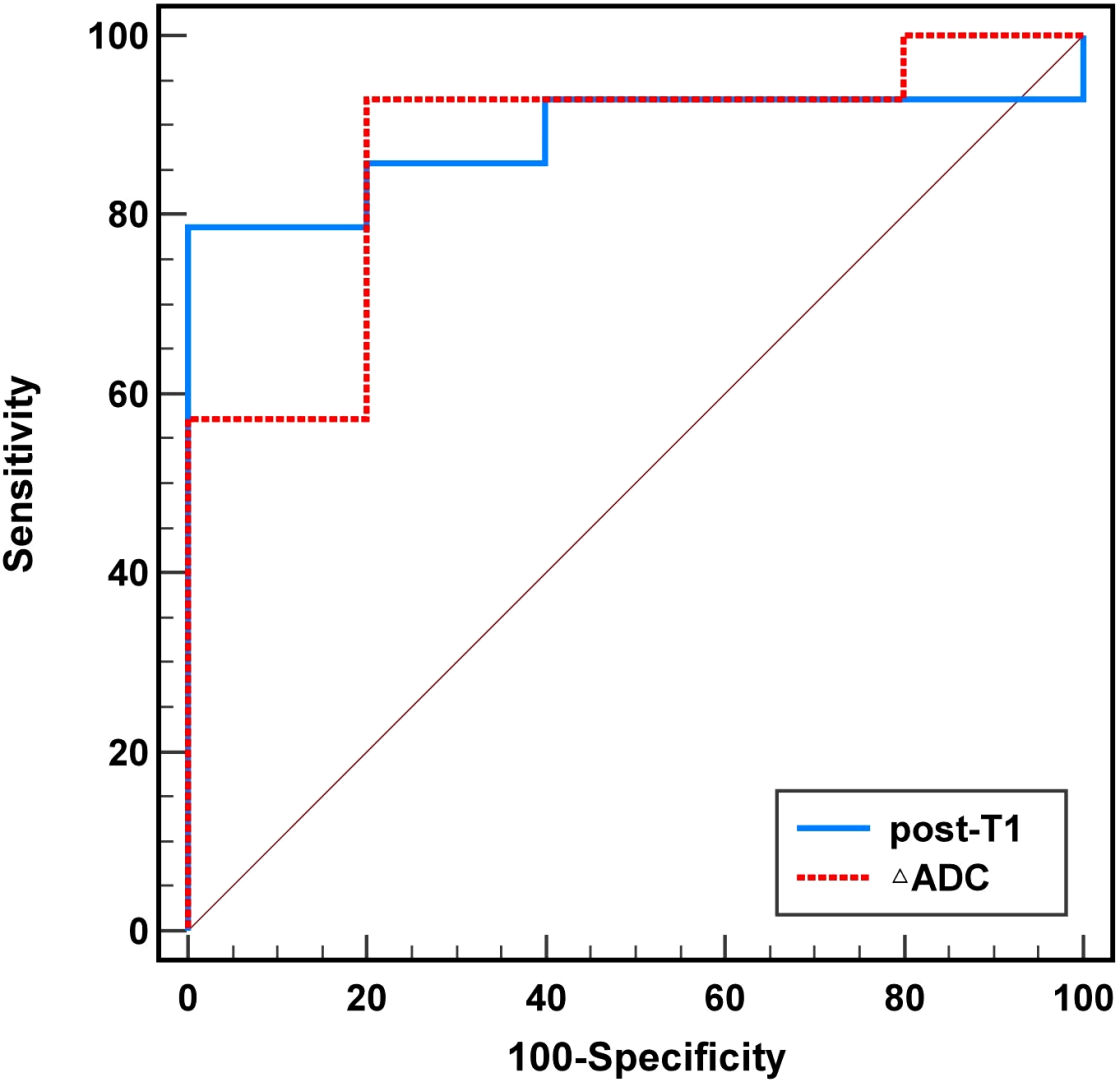
ROC curves of the post-treatment T1 and ΔADC values in predicting the efficacy of immunotherapy.

The ROC curve constructed with the post-treatment T1 values showed an AUC value of 0.886 (95% CI: 0.729-1.000), with a sensitivity of 1.000, a specificity of 0.786, and a cutoff value of 1,172.5 ms. Meanwhile, the ROC curve constructed with ΔADC values showed an AUC value of 0.871 (95% CI: 0.684-1.000), with a sensitivity of 0.800, a specificity of 0.929, and a cutoff value of -19.45×10^–6^ mm^2^/s (**Table 5**, **Figure 3**).

**Table 5:** Performance of the post-treatment T1 and ΔADC values in predicting the efficacy of Immunotherapy.

Parameters	AUC (95% CI)	Accuracy	Sensitivity	Specificity	PPV	NPV	Cut off
post-T1	0.886 (0.729-1.000)	0.842	1.000	0.786	0.625	1.000	1,172.5 ms
ΔADC	0.871 (0.684-1.000)	0.895	0.800	0.929	0.800	0.929	-19.45×10^–6^ mm^2^/s

AUC, area under the curve; 95% CI, 95% confidence interval; PPV, positive-predictive value; NPV, negative-predictive value.

## 4 Discussion

Immunotherapy, a therapeutic approach that activates the patient’s immune system to combat cancer cells, has achieved remarkable advancements in the management of advanced lung cancer over recent years. The accurate prediction of PD-L1 expression and the assessment of immunotherapy efficacy are of great significance for formulating personalized treatment strategies and enhancing patient prognosis in lung cancer. With its sophisticated imaging technology and sensitivity to microscopic tissue characteristics, MRI is capable of detecting pathological alterations within lesions at the molecular level prior to any visible morphological changes. This facilitates early and precise evaluations of immunotherapy efficacy, aligning with the contemporary development trend of lung cancer treatment. This study found that it is feasible to monitor the dynamic variations in T1 mapping and IVIM quantitative parameters during immunotherapy for effectively evaluating PD-L1 expression levels and treatment efficacy in advanced lung cancer.

T1 mapping is a quantitative magnetic resonance technique capable of acutely detecting subtle alterations in tissues, including water molecules, proteoglycans, collagen content, and other substances. The longitudinal (T1) relaxation time within the image voxel serves as a comprehensive indicator reflecting the tissue characteristics of organisms. With the development of rapid imaging techniques, T1 mapping has been widely studied and applied in various disease fields, including cardiology, hepatology, and nephrology (14–16). Similarly, T1 mapping has demonstrated high predictive value in the assessment of lung diseases (17, 18) and differentiating histological types of lung cancer (10–12). A study by Li et al. (10) further revealed a positive correlation between the T1 value and Ki-67 proliferation index, indicating that the metabolism and proliferation of tumor cells alter their adjacent microenvironment, thereby influencing the T1 value. This observation holds potential for enhancing clinical evaluations of disease progression in lung cancer patients (19, 20). In addition, IVIM represents a combination of several different b-values from low to high. Multiple technical indicators, including ADC, D, D*, and f values, are obtained utilizing the bi-exponential model. Specifically, the ADC value serves as an indicator for evaluating the diffusion degree of water molecules within tissue cells; the D value reflects the actual diffusion of water molecules within the region of interest; the D* value is the pseudo-diffusion coefficient, reflecting the diffusion effect within the voxel due to microcirculatory perfusion; and the f value represents the ratio of perfusion effect to total perfusion effect due to microcirculation (21). Numerous previous studies have demonstrated the correlations between IVIM quantitative parameters and critical tumor characteristics such as tumor cell density, proliferation index, and the density of surrounding microvessels (22–24). T1 mapping and IVIM technology are expected to provide more comprehensive and accurate information for the diagnosis and treatment evaluation of tumors.

However, the value of T1 mapping and IVIM in predicting PD-L1 expression and assessing immunotherapy efficacy in lung cancer has not been elucidated. The results of this study showed that the T1 value of the positive PD-L1 expression group was higher than that of the negative group. This discrepancy can be attributed to the heightened immunosuppression in the positive PD-L1 expression group, characterized by the infiltration of macrophages, lymphocytes and other immune cells around the malignant tissues. Higher tissue proton density leads to prolonged T1 relaxation time of tumor tissues. As in the findings of Bortolotto et al. (11), there was no statistical difference in the T1 values between the two groups. In another study utilizing IVIM quantitative parameters to assess PD-L1 expression in non-small cell lung cancer (NSCLC) patients, it was observed that the ADC and D values in the positive PD-L1 expression group were higher than those in the negative group, and the f value was lower than that in the negative group (25). Furthermore, the D* value exhibited a significant decrease, with a mechanism that contemplates an inverse correlation between the D* value and inflammatory infiltration. Nevertheless, this study indicated that the ADC, f, and D* values in the positive PD-L1 expression group were higher than those in the negative group, and the D value was lower than that in the negative group; however, these differences were not statistically significant.

Beyond the limited sample size that compromises statistical power, the high heterogeneity of lung cancer, encompassing histopathological subtypes, tumor differentiation grades, and key genetic mutations, may increase intra-group data variability. Additionally, the association between MRI quantitative parameters and PD-L1 expression is regulated by multiple complex biological processes, such as immune cell infiltration and tumor angiogenesis, which may contribute to the inconsistencies observed in current studies. Therefore, the relationship between MRI quantitative technology and PD-L1 expression still requires further investigation and verification.

In this study, we observed a significant reduction in the T1 value within the effective group after immunotherapy, which was significantly lower than that of the ineffective group. This alteration could be attributed to a combination of factors such as the death of tumor cells, the attenuation of inflammatory response, and the alteration of tissue structure. Consequently, T1 mapping is expected to be a promising biomarker for evaluating the efficacy of immunotherapy in lung cancer. The results of this study also showed that the ADC, f, D, and D* values were elevated after immunotherapy in the effective group, with a significant increase in ADC values. Conversely, the D value of the ineffective group increased slightly, and the ADC, f, and D* values decreased slightly, but the differences were not statistically significant. Comparison between the two groups demonstrated a significant difference in ΔADC values (*p* = 0.031), suggesting a reduction in tumor cell density, nucleoplasmic ratio, as well as immature blood vessels following immunotherapy. And the expansion of the extracellular gap increased the space for the free diffusion of water molecules. This finding is consistent with the results of several previous studies, which showed that the ADC value after treatment for lung cancer was significantly higher than that before treatment (26–28); Bao et al. (27) also confirmed that the ΔADC values in the pathological complete response (pCR) group were significantly higher than those in the non-pCR group after neoadjuvant chemo-immunotherapy (NCIT). Our analysis revealed that the f, D, and D* values changed in the effective group treatment, but there was no statistical difference between the two groups. Given the variability in the diffusion degree of water molecules among different pathological types of lung cancer, it may also be attributed to the inflammatory response within the tumor tissues that caused vasodilation and enhanced permeability, consequently leading to increased perfusion-related parameters. The above results not only deepen our understanding of the mechanisms of immunotherapy in lung cancer, but also provide important practical guidance for subsequent research and clinical applications.

This study has certain limitations. Due to the poor compliance of patients with advanced lung cancer, collecting data poses significant challenges. Consequently, it is necessary to employ large-sample, multicenter research designs and establish independent validation cohorts to further validate the predictive efficacy and generalization capabilities of MRI quantitative parameters. Additionally, MRI examination takes a long time and is susceptible to image distortion caused by patients’ respiratory movements; thus, optimizing the scanning sequence is essential for improving the image quality.

## 5 Conclusion

In summary, through dynamic monitoring T1 mapping and IVIM quantitative parameters, we were able to accurately evaluate the efficacy of immunotherapy in patients with advanced lung cancer. Notably, T1 and ADC values demonstrated high predictive value, which is expected to provide a quantitative evaluation basis for judging the patients’ response to immunotherapy and optimizing the treatment strategies.

## Data Availability

The raw data supporting the conclusions of this article will be made available by the authors, without undue reservation.
